# Neural mobilization reverses behavioral and cellular changes that characterize neuropathic pain in rats

**DOI:** 10.1186/1744-8069-8-57

**Published:** 2012-07-29

**Authors:** Fabio M Santos, Joyce T Silva, Aline C Giardini, Priscila A Rocha, Arnold PP Achermann, Adilson S Alves, Luiz RG Britto, Marucia Chacur

**Affiliations:** 1 Department of Anatomy, Laboratory of Functional Neuroanatomy of Pain, Sao Paulo, Brazil; 2 Department of Physiology and Biophysics, Laboratory of Cellular Neurobiology, Institute of Biomedical Sciences, University of São Paulo, Sao Paulo, Brazil; 3 Professor of Anatomy from University Nine of July, Sao Paulo, Brazil; 4 Laboratory of Functional Neuroanatomy of Pain Department of Anatomy Institute of Biomedical Sciences, University of São Paulo, Av. Prof. Lineu Prestes, Sao Paulo, 2415 05508-900, Brazil

**Keywords:** Sciatic nerve, DRG, Hyperalgesia, Satellite cells, NGF, Pain

## Abstract

**Background:**

The neural mobilization technique is a noninvasive method that has proved clinically effective in reducing pain sensitivity and consequently in improving quality of life after neuropathic pain. The present study examined the effects of neural mobilization (NM) on pain sensitivity induced by chronic constriction injury (CCI) in rats. The CCI was performed on adult male rats, submitted thereafter to 10 sessions of NM, each other day, starting 14 days after the CCI injury. Over the treatment period, animals were evaluated for nociception using behavioral tests, such as tests for allodynia and thermal and mechanical hyperalgesia. At the end of the sessions, the dorsal root ganglion (DRG) and spinal cord were analyzed using immunohistochemistry and Western blot assays for neural growth factor (NGF) and glial fibrillary acidic protein (GFAP).

**Results:**

The NM treatment induced an early reduction (from the second session) of the hyperalgesia and allodynia in CCI-injured rats, which persisted until the end of the treatment. On the other hand, only after the 4^th^ session we observed a blockade of thermal sensitivity. Regarding cellular changes, we observed a decrease of GFAP and NGF expression after NM in the ipsilateral DRG (68% and 111%, respectively) and the decrease of only GFAP expression after NM in the lumbar spinal cord (L3-L6) (108%).

**Conclusions:**

These data provide evidence that NM treatment reverses pain symptoms in CCI-injured rats and suggest the involvement of glial cells and NGF in such an effect.

## Background

The neural mobilization technique (NM) is a manual therapy method used by physiotherapists to treat patients with pain of neural origin, such as the compression of the sciatic nerve. NM is a noninvasive technique that has been effective in improving the quality of life of patients with neuropathic pain [1]-3]. In the literature, there are several studies indicating that NM is clinically effective against neuropathies [2,3]. However, the literature is scarce when surveyed on the possible mechanisms involved. There are only a few articles showing that joint mobilization it is able to decrease pain sensitivity in animals induced by neural injury or by capsaicin injection in to ankle joint [4-6].

During the execution of body movements, the nervous tissue protects the axons from tension and compression forces incident on peripheral nerves [7]. It should be stressed that the propagation of electrical impulses depends also on the elasticity of normal nervous tissue, as a normal component of its neurodynamics. The NM technique aims to restore mobility and elasticity of the peripheral nervous system and thus to improve the conditions of patients with various neural injuries [8-11].

Numerous studies have demonstrated that glial cells, both microglia and astrocytes, play a prominent role in the nociceptive pathways. After a threshold stimulus, activated glial cells release inflammatory mediators such as cytokines, prostaglandins, neurotrophic factors, ATP, NO, and glutamate [12]. These inflammatory mediators play a critical role in the development and maintenance of central sensitization and hyperalgesia. Furthermore, activated astrocytes have been shown to be a source of nerve growth factor (NGF) after injury [13] or after inflammatory cytokine application in vitro [14]. There is evidence that glial cells interact via neurotrophins with neurons and express NGF [15,16].

NGF belongs to a still growing family of neurotrophic molecules collectively called neurotrophins and that play an important role on survival, growth and neural differentiation [17]. NGF is produced and released by target tissues and uptaken in a retrograde way to maintain neuronal survival. Herzberg et al. (1997) suggested the involvement of NGF in the development of hyperalgesia based on by the increase of mRNA coding for NGF in both the sciatic nerve and in the DRG after constriction of the sciatic nerve [18]. Neurotrophins also play important roles in chronic pain states caused by nerve injury and inflammation [15,16,19]. In addition, neurotrophic factors released by activated microglia have been reported to generate a restoration of locomotor function after LPS-induced inflammation [20].

The aim of our study is to verify if NM can improve the pain-related behavior of rats after chronic constriction injury (CCI). In addition, we attempted to identify the possible changes of astrocytes, satellite cells and NGF in the spinal cord and DRG of adult neuropathic rats after NM.

## Results

### Effect of NM on mechanical and thermal hyperalgesia and allodynia induced by CCI

**Figure 1:**
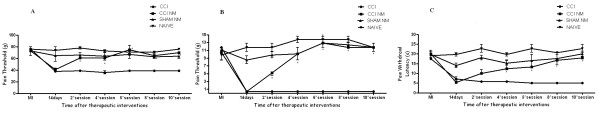
** Effect of neural mobilization on pain threshold induced by CCI in rats.** Pain thresholds as measured by the test of Randall & Selitto **(A)**, von frey test **(B)**, expressed in grams, and thermal hyperalgesia, assessed by Hargreaves test **(C)**, expressed in seconds. Measurements were determined before (base line- BL), 14 days after and at different intervals after NM sessions. The results represent the mean ± SEM of 10 animals per group. * P <0.05 for comparison between groups: CCI and CCI NM.

Rats submitted to CCI developed a protective behavior characterized by guarding of the ipsilateral hindpaw, which is a sign of neuropathic pain. The CCI induced a significant decrease of pain threshold (Figure 1A-C). These alterations were detected as early as day 1 and persisted for at least 1 month after surgery. The effects of the manipulation resulted in modifying pain thresholds (Figure 1). A decrease of mechanical hyperalgesia and allodynia was observed starting in the second session of neural mobilization (CCI-NM) when compared to CCI animals that did not receive NM. This effect remained constant for the next eight sessions (Figure 1 A,B). On the other hand, thermal hyperalgesia was inhibited only after the fourth NM session, an effect that remained constant until the last session (Figure 1C).

No significant difference of pain threshold was observed in sham-operated animals that received NM sessions (taken as control - sham-NM) during the whole period analyzed (Figure 1), and no significant differences of paw withdrawal threshold were observed on the contralateral side of injury between measurements or at any time post-lesion (data not shown). It is important to point that all CCI animals that received NM sessions exhibit a behavioral improvement, such as reduced guarding behavior and recovery of limping. No difference was observed in Naive or sham animals.

### Effects of NM on NGF and GFAP expression

**Figure 2:**
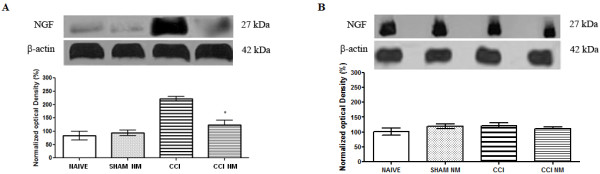
** Densitometric analysis of NGF protein levels in the DRG (A) and spinal cord (B).** The normalized average between sham and experimental groups (CCI) is reported. Data for naive animals were taken as 100%. Data are reported as mean ± SEM of 6 animals per group. * P <0.05 compared to CCI animals.

A single NGF-positive band was observed in DRG and spinal cord extracts from all groups analyzed. Figure 2 shows an increase of NGF protein levels on the ipsilateral side from experimental animals in the DRG (CCI and CCI NM) and spinal cord when compared to the same side from naive rats, taken as controls and designated as 100%.

Regarding DRG, densitometric analysis revealed an increase of NGF expression in animals with CCI (111% above the control) when compared to naive animals and a return to control levels in rats that received NM treatment (CCI-NM) (Figure 2A). No statistical differences of NGF expression were observed between sham- NM and naive or between sham and sham-NM animals (data not show). On the other hand, no difference was observed when the lumbar spinal cord was analyzed (Figure 2B).

**Figure 3:**
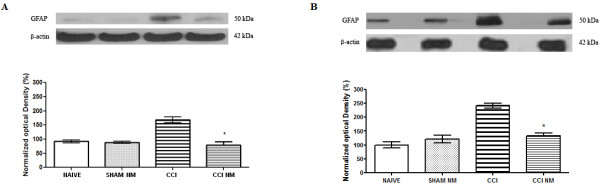
** Densitometric analysis of GFAP protein levels in the DRG (A) and spinal cord (B).** The normalized average between sham and experimental groups (CCI) is reported. Data for naive animals were taken as 100%. Data are reported as mean ± SEM of 6 animals per group. * P <0.05 compared to CCI animals.

Similar results were observed when we analyzed the GFAP-positive band in the DRG and lumbar spinal cord tissue described above. Our results showed an increase of GFAP levels (68% for DRG and 108% for spinal cord, above the control) after CCI injury when compared to naive animals and a return to normal levels after NM treatment (CCI-NM) (Figure 3). No difference was observed between naive and sham-NM or between sham and sham-NM (data not show). No differences were observed for β-actin between control and experimental sides at any of the time points tested (Figures 2 and 3).

### Double-labeling immunofluorescence for NGF and GFAP in the DRG after NM in neuropathic rats

**Figure 4:**
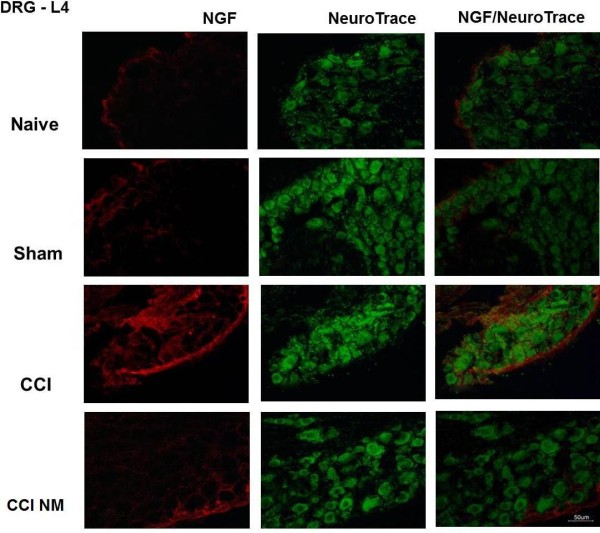
** Double-labeling of NGF and NeuroTrace in the ipsilateral DRG after CCI injury.** Naive, Sham (control), CCI (14 days after injury) and animals treated with neural mobilization (CCI NM) were analyzed. Double immunofluorescence shows NGF (red) and NeuroTrace (green). Note that NGF expression after neural mobilization decreased when compared to CCI. Scale bar, 50 μm.

**Figure 5:**
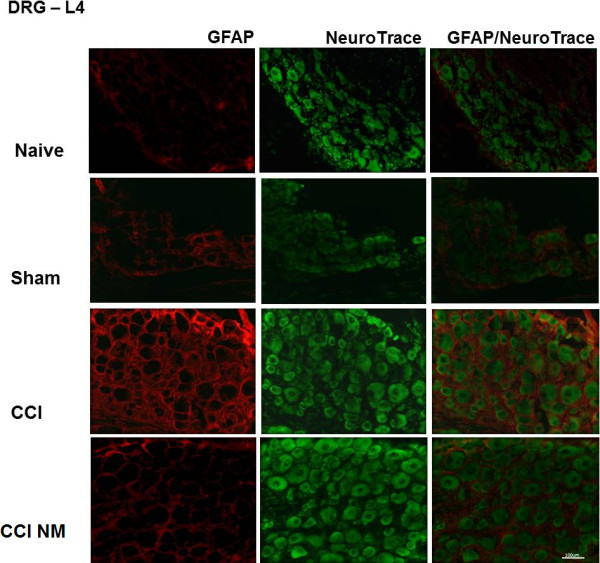
** Double-labeling of GFAP and NeuroTrace in the ipsilateral DRG after CCI injury.** Naive, Sham (control), CCI (14 days after injury) and animals treated with neural mobilization (CCI NM) were analyzed. Double immunofluorescence shows GFAP (red) and NeuroTrace (green). Note that GFAP expression after neural mobilization decreased when compared to CCI. Scale bar, 100 μm.

We also examined the immunostaining for NGF (Figure 4) and GFAP (Figure 5) in the lumbar (L4) DRG from CCI, sham and naive rats. Immunofluorescence was performed in the ipsilateral (right) sides of the CCI animals (with or without NM) and compared to the ipsilateral side of sham-operated rats or the same side naive animals (taken as controls).

A moderate number of cells labeled for NGF-immunoreactivity (NGF-IR) were observed in the DRG of naive and sham rats (Figure 4). After CCI, there was a marked increase in the number of NGF-IR cells in the ipsilateral side. We observed a decrease of NGF-IR in animals that received NM treatment in relation to untreated rats (Figure 4).

Regarding GFAP, we observed an increase of cells labeled for GFAP-IR in the DRG after CCI when compared with naive animals and a reduction after NM treatment when compared to CCI animals (Figure 5).

## Discussion

The aim of our study was to evaluate the effects of NM in behavioral improvement in neuropathic rats, and to evaluate NGF and GFAP levels in the DRG and spinal cord after NM.

Behavioral data confirmed the onset of neuropathic pain syndrome 3 days after CCI, which stabilized by the 14th day after injury, as shown by reduced mechanical, thermal and allodynic thresholds in the ipsilateral side of injury. We demonstrated those ten NM sessions each other day restored mechanical, thermal and allodynic sensitivity, as well as all behavior parameters that were associated with the neuropathic condition.

There is a growing body of evidence that a unilateral nerve injury induces contralateral changes. The effect of the unilateral peripheral nerve lesion on contralateral non-lesioned structures was reviewed by many authors. In general, the responses to contralateral injuries are usually in smaller magnitude and with a briefer time course compared to ipsilateral changes [21,22]. However, our results demonstrated an effect at the unilateral nerve injury which corroborates with others studies showing the same response [23-27]. Methodological variables could account for this discrepancy, such as different strains of rats, different methodologies or different suture material used for different authors [28].

The impact of halothane anesthesia does not interfere with behavior responses, since the nociceptive experiments were realized on subsequent day after NM sessions. In addition, studies demonstrated that from inhalation anesthetics, halothane was able to decrease nociceptive reactions (returned to basal levels) faster than other inhalatory anesthesics (enflurane, isoflurane, and desflurane) [29]. Furthermore, studies have shown that the use of inhalational anesthetic during nine sessions of physical therapy in rats did not change expression of glial cells markers [4]. Suggesting that this anesthetic does not influence in chronic models of sensitization [30,31].

Our behavioral data are in agreement with studies showing that NM is efficient as a therapeutic approach in clinical applications [1,3,11]. Studies realized recently, demonstrated the reversion of hyperalgesia, as well as nerve regeneration and decreased of glial cells expression such as microglia and astrocytes in the spinal cord, after joint mobilization in a model of sciatic nerve crush injury [4]. In addition, Malisza, K.L. et al. (2003) examine neuronal activation in the spinal cord due the secondary hyperalgesia resulting from intrajoint capsaicin injection [32].

On the other hand, studies realized by Vicenzino et al. (1998) and Sterling et al. (2001) using spinal manual therapy, showing a hypoalgesia effect but no influence on thermal hyperalgesia, suggesting an effect in inhibitory pathway from the dorsal periaquedutal gray area [33,34].

In this regard, we also evaluated the levels of NGF and GFAP in the DRG and spinal cord after NM treatment in CCI-injured animals. We demonstrated an increase of NGF expression in CCI-injured rats, and a decrease to normal levels after NM treatment in DRG tissues. On the other hand, no difference was observed in lumbar spinal cord tissue analyzed. This indicates that the increase of NGF occurs at time points that are coincident with its role in pain-related behaviors, suggesting that NGF is involved in the nociceptive effects induced by neuropathic pain. Our data are in agreement with several studies showing that NGF injections by systemic and peripheral routes induce nociception [15-17,19]. Indeed, NGF seems to be a critical mediator of all types of pain, including short-term pain [35], surgical pain [36], inflammatory pain [37] visceral pain [38] and neuropathic pain [19,38].

The pain induced by peripheral inflammation or neuropathic pain is currently being linked to the involvement of glial cells or satellite cells located in the spinal cord and DRG, respectively [39,40]. Our studies demonstrate through Western Blot technique an increase in optical density of the bands marked with astrocytes and satellite cells (GFAP) in spinal cord and DRG, respectively, in animals with chronic constriction sciatic nerve (CCI). Animals with CCI and treated with NM, we observed a decrease in GFAP-IR in both tissue analyzed and a reversal of hyperalgesia and allodynia. Our findings corroborate Garrison et al. 1991 showed an increased density of these cells, specifically astrocytes, in the spinal cord after induction of ligatures in the sciatic nerve [41]. Nerve injury activates glial cells, which produce and release inflammatory mediators that act on glial cells and neurons, sensitizing the dorsal horn and facilitating pain transmission [39,42]. Here we demonstrated an increase of NGR-IR and GFAP-IR in DRG neurons after CCI-injury, which was decreased after NM treatment. This suggests an involvement of NGF and glial cells in our experimental model, which remains to be fully understood. Our observations were generally consistent with the literature regarding the time course and localization of astrocytes and satellite cells activation [43]. Nerve injury and inflammation have been shown to lead to proliferation and hypertrophy of satellite cells, and to upregulate other molecules, including neurotrophins, tumor necrosis factor α, and functional gap junctions [17,43].

Glial cells appear to be a pivotal component of the neuroinflammatory process, and it is known that this type of cell, in the spinal cord, reacts to peripheral nerve injury and inflammation by activation, proliferation, and by releasing messengers that contribute to pathological pain. Glial cells participate in synaptogenesis and maintenance of synaptic activity and expression of receptors, transporters, ionic channels, at least through the release of neurotrophins such as NGF [44-46].

In summary, our data reveal a reversal of the NGF and GFAP increase in DRG by NM treatment in CCI animals. On the other hand, we observed the involvement of GFAP only in spinal cord. We also demonstrated that NM sessions are able to improve behavioral responses, providing evidence of the importance of NM in restoring the changes caused by CCI injury, which contribute to neuropathic pain behavior. These findings suggest that NM reverses the nociceptive behavior of neuropathic animals, and suggest the involvement of glial cells and NGF in such an effect.

## Methods

### Animals

Male Wistar rats, weighing between 180 and 220 g (ca. 2 months-old) were used in all experiments. They were singly housed and maintained on a 12:12 h light/dark cycle. The rats were adapted to the experimental environment three days before the experiments started. All animals were tested during the light cycle at the same time of the day (9:00 am–12:00 a.m.). All procedures were approved by the Institutional Animal Care Committee of the University of São Paulo (protocol number 26 – book number 02/2010). Efforts were made to minimize the number of animals used and their suffering.

### Surgical procedure

#### Chronic constriction injury - (CCI)

For the induction of neuropathic pain, chronic constriction of the sciatic nerve was performed as described by Rats were anesthetized with halothane (Cristalia, Brazil) [47]. The common sciatic nerve was exposed at the level of the middle of the thigh by blunt dissection through the biceps femoris. Proximal to the sciatic trifurcation (about 7 mm), the nerve was freed of adhering tissue and 4 ligatures (4.0 chromic gut- Paramed) were tied loosely around it with about 1 mm spacing. Great care was taken to tie the ligatures, such that the diameter of the nerve was seen to be just barely constricted. The incision was closed in layers. In sham-operated rats the sciatic nerve was exposed but left unaffected and served as a control. Each rat was closely observed during the recovery from anesthesia and then returned to the home cage and was observed carefully during the following 24 hours. During the 5 day-period after CCI the walking and cage exploration, degree of limping, and conditions of the hindpaw, including signs of excessive grooming or autotomy, were all closely observed.

### Neural Mobilization Technique – (NM)

The NM technique used has been described by Butler (1989). Briefly, to perform the technique rats were anesthetized with halothane with a continuous flow of medicinal oxygen throughout the procedure (5 ml/L) [48]. After anesthesia, the animals were positioned in the left lateral position to mobilize the right side (ipsilateral to CCI). The right knee joint was then positioned in full extension (at 0 degrees) and remained so throughout the session. Moreover, the right hip joint was bent between 70 to 80 degrees with the knee in extension until obtaining a small resistance induced by stretching the muscles from compartimentum posterius femoris (M. biceps femoris, M. semimembranosus and M. semitendinosus). After the therapist fell the resistance, the angle of this joint it is interrupted. At this time the ankle joint, will be angled between 30 to 45 degrees, using the same principle, as described before. After all joints are positioned with minimal resistance of those muscles, oscillatory movements are initiated. The right ankle joint was manipulated in dorsi-flexion (30 to 45 degrees) by approximately 20 oscillations per minute during 2 minutes, followed by a 25-second pause for rest. The treatment took place for ten minutes, and in the last minute the cervical spine was fully flexed, with the purpose of tensioning the entire neuraxis [49]. Treatment with the NM technique started 14 days after injury or sham animals, and the NM sessions was applied every other day, for a total of 10 sessions.

### Behavioural experiments

The development of neuropathic pain symptoms in the hindpaw was evaluated by testing mechanical (Randall and Selitto test) and thermal hyperalgesia (Hargreaves test) [50,51] and allodynia (Von Frey hair test). Those sensory tests were used in light of literature data showing that CCI induces both mechanical and thermal hyperalgesia and allodynia of the ipsilateral hindpaw [52].

Paw withdrawal was measured before surgery (Baseline-BL), 14 days after surgery (to confirm neuropathic pain), and intercalated with NM sessions to avoid the impact of halothane anesthesia on subsequent pain testing. All behavioral tests were double-blind.

#### Mechanical hyperalgesia (Randall and Selitto test)

An Ugo-Basile pressure apparatus [51] was used to assess pressure pain thresholds prior to surgery and again at different times thereafter. Testing was blind in regard to group designation. Briefly, increasing force (16 g/s) was applied to the right hindpaw. The force needed to induce paw withdrawal was recorded as the pain threshold. To reduce stress, the rats were habituated to the testing procedure the day before the experiment.

#### Thermal hyperalgesia (Hargreaves test)

The Hargreaves test, which measures response latencies to hindpaw thermal stimulation [50] was used. Each rat was placed in a clear plastic cylinder (24 cm *H* × 16.5 cm *D*) atop a glass floor in a temperature controlled room (28 ± 1°C) and allowed to adapt to this environment for 30 min before testing. Rats were first habituated to the experimental context (room and apparatus) for 2 consecutive days for 45 minutes/day. BL paw withdrawal values were calculated from an average of two to three consecutive withdrawal latencies of the right hindpaw measured at 10-min intervals. Voltage to the light source was adjusted to yield BL latencies ranging 15–20 s. A cut-off time of 40 s was imposed to avoid tissue damage.

#### Low threshold mechanical allodynia (von Frey test)

The von Frey test [53] was used to assess low-threshold mechanical pain thresholds prior to surgery and again at different times thereafter. Testing was blind in regard to group designation. This test was performed as previously described [54]. Briefly, a logarithmic series of 10 calibrated Semmes-Weinstein monofilaments (von Frey hair test, Stoelting, USA) were applied to the middle of the plantar surface of the right hindpaw, for a maximum of 10 s to determine the threshold intensity of the stiffness stimulus required to elicit a paw withdrawal response. Log stiffness of the hairs is determined by ranges starting at 3.61 N (0.407 g); (3.61 (0.407 g.); 3.84 (0.692 g.); 4.08 (1.202 g.); 4.17 (1.479 g.); 4.31 (2.041 g.); 4.56 (3.630 g.); 4.74 (5.495 g.); 4.93 (8.511 g.); 5.07(11.749 g) and reaching 5.18 (15.136 g.). Baseline assessment was initiated with the 2.041-g hair. In the event of paw withdrawal, the same hair was again presented 60–90 s later. If the response was elicited again, the 0.407 g monofilament was presented. In the absence of a paw withdrawal response to the 0.407 g stimulus, the next stronger monofilament was presented (0.692 g). The monofilament that elicited a clear response was recorded, and was presented once again 30–60 s later. If the animal withdrew its paw in two consecutive trials with the same stiffness value, no further von Frey hairs were tested. However, in the absence of a response to the initial 2.041-g monofilament, presentation of monofilaments continued in ascending order until two consecutive responses were elicited from the same monofilament. All single responses were recorded, but assessment was complete only after two consecutive responses to the same stimulus. Failure to respond to the strongest stimulus (15.136 g) was considered to be the cut-off value. Response to the weakest stimulus (0.407 g) was considered to be the lower cut-off value. To reduce stress, rats were adapted to the experimental environment daily for 3 days before the experiments.

### Immunoblotting

Western blotting analyses were performed on samples from individual animals. Neuropathic (CCI), Sham and naive rats were sacrificed by decapitation under light halothane anesthesia and the ipsilateral DRG (from L3-L6) and lumbar spinal cord (L3-L6) were quickly removed and homogenized in an extraction buffer containing 100 mM Tris, pH 7.4, 10 mM EDTA, 2 mM PMSF, and 10 μg/mL aprotinin. After extraction, the homogenates were centrifuged at 11.5 *g* for 20 min and the protein concentration of the supernatant was determined using the Bradford protein assay with albumin as a standard (Bio-Rad, USA) [55]. Samples containing 75 μg protein were loaded on a 12% acrylamide gel and electrotransferred to nitrocellulose membranes using a Bio-Rad miniature transfer apparatus during 1.5 h at 120 V. After transfer, the membranes were treated for 2 h at room temperature with a blocking solution containing 5% powdered milk, washed and incubated overnight at 4°C with rat monoclonal antibodies against rat NGF (F30, 1:1000; Santa Cruz Biotechnology, INC) (Wu et al., 2007) and against mouse GFAP (1:1000; Sigma, St. Louis, MO) (Chacur et al., 2004b) . The membranes were then washed and incubated for 2 h at room temperature with a peroxidase-conjugated, anti-rat secondary antibody, diluted 1:5000 (ZIMED Laboratories Inc) and an anti-mouse secondary antibody (GE Healthcare, USA). β-actin was used as an endogenous control (1:10000, Sigma). The specifically bound antibody was visualized using a chemoluminescence kit (Amersham Biosciences). The blot was analyzed densitometrically using NIH-Scion Image 4.0.2 and quantified by optical densitometry of the developed autoradiographs (Scion Corporation, USA).

### Immunofluorescence and image analysis

After the appropriate survival time, the animals were deeply anesthetized with ketamine (0.1 mL/kg) and xylazine (0.1 mL/kg) and perfused through the heart with phosphate-buffered saline and 4% paraformaldehyde in 0.1 M phosphate buffer (PB), pH 7.4. The DRG from the lumbar spinal cord (L4) were removed and post-fixed for 4 h in 4% paraformaldehyde. The DRG were transferred to a 30% sucrose solution in PB to ensure cryoprotection, which lasted for 48 h, and transverse sections of DRG were performed by embedding it in OCT compound, freezing and then cutting (12 μm-sections) on a cryostat. DRG sections were incubated with a mouse monoclonal antibody against NGF (Santa Cruz Biotechnology, INC) and GFAP (Sigma, St. Louis, MO) diluted 1:1000 in PB containing 0.3% Triton X-100 and 5% normal goat serum. Incubations with the primary antibody were conducted overnight at 24°C; sections were then washed three times for 10 min each in PB and incubated with a tetramethyl rhodamine isothiocyanate - TRICT – conjugated affinipure goat anti-mouse igG (Jackson ImmunoResearch , USA) diluted 1:100 in PB for 2 h at 24°C. After several washes in PB, the same sections were incubated with Neurotrace (500/525 green fluorescent Nissl stain, Molecular Probes) at 1:1000 in PB during 30 minutes for neuronal identification. Controls of the experiments consisted of the omission of primary antibodies, and no staining was observed in these cases. After washing, the tissue was mounted using VectaShield (Vector Laboratories, Burlingame, CA).

Slides were analyzed in a fluorescence microscope coupled to a digital camera. Figures were mounted with Adobe Photoshop CS. Manipulation of the images was restricted to threshold and brightness adjustments of the whole image. It should be stressed that the antibodies used here has been extensively tested and characterized [26,56-58]. Regarding immunofluorescence analyzes, we used as qualitative test, the immunoblotting assays was used as quantitative test.

### Statistical analysis

Data were statistically analyzed by analysis of variance (ANOVA) and sequential differences between means were tested by Tukey contrast analysis at P < 0.05 [59].

## Competing interests

All authors declare that they have no competing interests.

## Authors’ contributions

Fabio M. Santos, Aline C. Giardini, Joyce T. Silva, carried out all experiments included in this article. Priscila A. Rocha and Arnold P P Achermann, help in the behavioral experiment, Adilson S. Alves, was a technique in this article, Dr. Luiz R.G. Britto help in draft the manuscript and discuss the results together with the students; Dr. Marucia Chacur, was a mentor of the project and corresponding author. Also, all authors read and approved the final manuscript.
